# *Celsr3* Inactivation in the Brainstem Impairs Rubrospinal Tract Development and Mouse Behaviors in Motor Coordination and Mechanic-Induced Response

**DOI:** 10.1007/s12035-022-02910-7

**Published:** 2022-06-09

**Authors:** Boli Chen, Fuxiang Li, Bin Jia, Kwok-Fai So, Ji-An Wei, Yuchu Liu, Yibo Qu, Libing Zhou

**Affiliations:** 1 Guangdong-Hongkong-Macau CNS Regeneration Institute of Jinan University, Key Laboratory of CNS Regeneration (Jinan University)-Ministry of Education, Jinan University, Huangpu Avenue West 601, Guangzhou, 510632 People’s Republic of China; 2 Neuroscience and Neurorehabilitation Institute, University of Health and Rehabilitation Sciences, Qingdao, 266071 Shandong People’s Republic of China; 3 Department of Neurology and Stroke Center, The First Affiliated Hospital & Clinical, Neuroscience Institute of Jinan University, Guangzhou, 510632 People’s Republic of China; 4 Bioland Laboratory (Guangzhou Regenerative Medicine and Health Guangdong Laboratory), Guangzhou, 510005 People’s Republic of China; 5 Co-Innovation Center of Neuroregeneration, Nantong University, Jiangsu, People’s Republic of China; 6 Guangdong-Hong Kong-Macao Greater Bay Area Center for Brain Science and Brain-Inspired Intelligence, Guangzhou, 510515 People’s Republic of China; 7 School of Biological Sciences, The University of Hong Kong, Hong Kong SAR, China

**Keywords:** Rubrospinal tract, Mechanical sensation, Celsr3, Brainstem, Axon projection

## Abstract

Inactivation of *Celsr3* in the forebrain results in defects of longitudinal axonal tracts such as the corticospinal tract. In this study, we inactivated *Celsr3* in the brainstem using En1-Cre mice (*Celsr3* cKO) and analyzed axonal and behavioral phenotypes. *Celsr3* cKO animals showed an 83% reduction of rubrospinal axons and 30% decrease of corticospinal axons in spinal segments, associated with increased branching of dopaminergic fibers in the ventral horn. Decreases of spinal motoneurons, neuromuscular junctions, and electromyographic signal amplitude of the biceps were also found in mutant animals. Mutant mice had impaired motor coordination and defective response to heavy mechanical stimulation, but no disability in walking and food pellet handling. Transsynaptic tracing demonstrated that rubrospinal axons synapse on spinal neurons in the deep layer of the dorsal horn, and mechanical stimulation of hindpaws induced strong calcium signal of red nuclei in control mice, which was less prominent in mutant mice. In conclusion, Celsr3 regulates development of spinal descending axons and the motor network in cell and non-cell autonomous manners, and the maturation of the rubrospinal system is required for motor coordination and response to mechanical stimulation.

**Supplementary Information:**

The online version contains supplementary material available at 10.1007/s12035-022-02910-7.

## Introduction

To fulfill their motor function, spinal motor neurons integrate multiple descending inputs from cortical and subcortical origin. Besides corticospinal neurons in the motor cortex, nuclei in the brainstem project to spinal segments, forming rubrospinal, vestibulospinal, and reticulospinal tracts, all of which participate in motor control and coordination [1].

The rubrospinal tract (RST) originates from large glutamatergic neurons in the magnocellular red nuclei in the basal midbrain. RST axons cross the midline in the ventral tegmental decussation and innervate contralateral spinal segments [2]. The cytoarchitecture and neural connectivity of red nuclei vary in different species, reflecting differences in motor control [3]. In rodents, the majority of RST axons synapse on spinal interneurons of intermediate Rexed’s laminae V and VI, and influence indirectly motor neuron activity. A small population make direct rubro-motoneuronal connections implicated in the control of distal forelimb muscles [4].

Development and maturation of the rubrospinal system are driven by intrinsic genetic programs and modulated by parallel descending axonal tracts. For example, inactivation of transcription factor *Pou4f1* impaired the expression of *Robo1* and *Slit2* in the midbrain and resulted in abnormal maturation of red nuclei and RST fasciculation deficits [5]. The RST and corticospinal tract (CST) are two major descending motor systems that largely work in concert and synapse on the same interneuron pool. The interruption of CST at early postnatal stages enhances the red nucleus motor map and RST projection area in the spinal cord [6], presumably by altering the balance between CST and RST. In mice with genetic absence of CST, the RST developed compensatory spinal projections presumably to compensate defective CST function [7].

Although the main role of descending tracts is to regulate motor function, recent studies showed that the CST is also implicated in tactile sensation [8], and that motor and sensory modulation commands are controlled by segregated axonal bundles within the CST [9]. That the RST could also be involved in sensory processing is supported by neuroimaging studies in humans [10] and by recording responses of neurons in the magnocellular red nucleus to painful stimuli [11]. However, the role of the RST in sensory modulation is not fully understood.

Genetic models provide tools to study the development, maturation, and function of axonal tracts. Atypical cadherin *Celsr3* is crucial for the development of various longitudinal axon bundles [12], and conditional inactivation of *Celsr3* in the cortex results in the absence of the CST [13]. In this study, we conditionally inactivated *Celsr3* in the brainstem to further understand its role in the development of descending axonal bundles, and to probe the effect of these neural circuits on spinal cord function.

## Materials and methods

### Animals

All experimental procedures were approved by the Laboratory Animal Ethics Committee at Jinan University. *En1-Cre;Celsr3*^±^ males were crossed with *Celsr3*^*f/f*^ females to generate *En1-Cre;Celsr3*^*f/−*^ conditional knockout mice (*Celsr3* cKO). *En1-Cre;Celsr3*^*f/*+^ or *Celsr3*^*f/−*^ littermates were used as controls. *En1-Cre* males were crossed with *Rosa26*^*GFP*^ females to monitor Cre expression. Male and female mice were used indiscriminately.

### Immunohistochemistry

Animals were anesthetized, and perfused intracardially with 4% paraformaldehyde (PFA) in 0.01 M phosphate-buffered saline (PBS). Brains, spinal cords, and biceps brachii were collected for preparing sections with a sliding microtome (Leica, Germany). Floating methods were used for immunostaining. Briefly, after washing with 0.01 M PBS plus 0.3% Triton, sections were blocked in 3% bovine serum albumin plus 10% normal donkey serum for 2 h, and incubated with the primary antibodies overnight at 4 °C. The primary antibodies included goat anti-choline acetyltransferase (ChAT; 1:500, AB144p, Millipore), rabbit anti-protein kinase Cγ antibody (PKCγ; 1:400, ab109539, Abcam), rabbit anti-tyrosine hydroxylase (TH; 1:1000, AB152, Millipore), rabbit anti-serotonin (5-HT; 1:1500, S5545, sigma), rabbit anti-NF200 (1:500, n4142, Sigma), and guinea pig anti-vGlut2 (1:1,000, AB2251, Millipore). After three rinses in 0.01 M PBS, signal was disclosed by secondary fluorescent antibodies (Alexa Fluor 488 or 546, 1:1,000, A11073/A11055/A21206/A10040, Thermo Fisher), and α-bungarotoxin conjugated to Alexa Fluor 546 (*α*-BT; 1:1,000, T1175, Molecular Probes) was used to label acetylcholine receptors.

### Non-*trans*-synaptic Tracing

Fluoro-Gold (FG) was used for retrograde non*-trans*-synaptic tracing. In anesthetized mice, 0.5 μl FG (6% in distilled H_2_O; 52–9400, Fluorochrome) was injected into right C6 spinal segment (650 μm to the midline and 600 μm in depth) using a glass capillary. One week later, brains were collected and sagittal sections were prepared to observe labeled neurons in the cortex and brainstem. To study propriospinal projections, CTB (0.5 μl; 0.5% in 1 × PBS; C22843, Invitrogen) was injected into C8–T1 segments on the right side (650 μm lateral to the midline, 600 μm in depth) with a glass capillary, and C3–C4 spinal segments were collected 7 days later. For anterograde tracing of rubrospinal or corticospinal projecting neurons, AAV9-CMV-GFP (1E + 13 μg/ml, 500 nl/injection; Vigene Biosciences Branch) was injected into red nuclei (− 3.5 mm to the bregma, ± 0.84 mm lateral to the midline, and 3.65 mm in depth) or unilateral motor cortex with 5 sites (1.5 mm lateral to the midline; − 1.0, − 0.5, 0, 0.5, and 1 mm anteroposterior to the bregma; 0.5 mm in depth) respectively. Sections of spinal cords or brains were prepared for imaging 3–4 weeks later.

### Transsynaptic Tracing

To trace spinal neurons directly synapsed by rubrospinal axons, scAAV1-hSyn-Cre virus (2.91E + 13 μg/ml, 500 nl/injection; Vigene Biosciences Branch) was injected into red nuclei (− 3.5 mm to the bregma, ± 0.84 mm lateral to the midline, and 3.65 mm in depth) in Ai14-tdTomato mice. Three weeks later, spinal cords were collected to prepare sections, and images were captured by confocal microscopy.

### Stimulation-Induced Neuronal Activity Recording

AAV2/9-hSyn-GCaMP6s virus (2.05E + 12vg/ml, 500 nl/injection; Brain Case) was injected into right red nuclei as described above. Three weeks later, optical fibers (Thinker Tech Nanjing Biotech) were implanted into the injection site, and were attached to the skull using dental cement. The calcium fluorescence signal in red nuclei was recorded using fiber photometry (Thinker Tech Nanjing Biotech), upon stimulating the plantar surface of hindpaws using a pin with inter-trial intervals of 30 s. The values of fluorescence change (Δ*F*/*F*) were calculated as the ratio of (*F* − *F*0)/*F*0, in which *F*0 was the averaged baseline fluorescence signal in red nuclei before the stimuli.

### Cell Count and Fiber Analysis

#### PKCγ-Labeled Corticospinal Fibers

C5-T1 spinal segments were cut into 6 series of transverse sections, one of which was used for anti-PKCγ immunofluorescent staining. PKCγ immunoreactive areas in the dorsal funiculus were measured using ImageJ and the average represented one sample. Four animals were used in each group.

#### ChAT-Positive Spinal Motoneurons

One series of spinal sections from C5–T1 segments (6 series/animal) were processed with anti-ChAT immunofluorescent staining, and ChAT-positive neurons in the ventral horn were counted and averaged as one sample. Five animals were used in each group.

#### FG-Labeled Neurons

Serial sagittal sections were prepared and labeled neurons were counted in the cortex, midbrain, and pons in every fourth section. Three animals were used in each group.

#### Fiber Reconstruction

C8–T1 spinal segments were cut into 6 series of transverse sections, and immunostained for TH or 5-HT. Images were scanned at 0.5-μm intervals for stack reconstruction using confocal microscopy (Zeiss, Germany), followed with fiber reconstruction using the “filament function” in the Imaris software (BitPlane AG, Switzerland). Three animals were used in each group.

### Electromyogram (EMG) Recording

Under anesthetization with propofol, musculocutaneous nerves and biceps were exposed under a stereomicroscope. A recording electrode was inserted into the biceps (posterior third, 0.2 mm in depth), and a stimulation (30 μA, 0.5 ms) was administered to the musculocutaneous nerve using a monopolar stimulation electrode. EMG signals were collected using a multi-channel system (VikingQuest EMG/EP System, Nicolet, USA). In each animal, the recording was repeated at least 6 times with an interval of 3 min, and the averaged result was taken as one sample.

### Behavioral Studies

Young adult animals (2–3 months old) were used for behavioral tests, which were carried out by examiners blind to mouse genotypes.

#### Open-Field Test

Animals were placed in a 50 × 50 × 35 cm transparent box and allowed to explore freely for 5 min, after which the walking traces were captured using a camera for 15 min. Data were analyzed using the EnthoVision XT 8.0 software.

#### Grip Strength Measurement

Forelimb grip strength was measured using a grip strength meter (Ugo Basile, Italy), 3 times (20-min interval/trial) in each animal, and the data were averaged for one sample.

#### Grid Walking Test

Animals were placed on a square frame with parallel horizontal bars (1-cm interval between bars) to explore freely for 3 min, and then the walking was videotaped for 50 steps. The percentage of footslips (forepaws missing/slipping the bar) was calculated.

#### Rotarod Test

Motor coordination and balance were evaluated using an accelerating Rotarod (Ugo Basile, Italy). After a 1-min adaption on the stationary rod, animals were subjected to the rotating rod with increasing speed from 2 to 40 rpm in 5 min. The average falling latency was calculated from 3 trials with a 20-min interval, to represent one experiment.

#### Sensation Tests

Laser heat pain was estimated using a plantar analgesia meter (Series8/Model 390, IITC Life Science). After a 2-h adaptation, a laser light stimulated paws and the withdraw latency was recorded. The average of 5 trials (2-min interval) in each animal was taken as one experiment. Thermal sensation was studied using the hot plate. The initial temperature started at 30 °C and gradually increased at 10 °C/min. The temperature when mouse hindpaws were withdrawn or licked was recorded. The average temperature of 3 trials (20-min interval) was taken as one sample. Mechanical sensation was evaluated using the von Frey test. The plantar surface of hindpaws was stimulated by a gradually increasing force with a series of von Frey filaments (0.02, 0.04, 0.07, 0.16, 0.4, 0.6, 1, and 2 g). Each filament was tested 10 times. Between individual measurements, von Frey filaments were applied at least 3 s after the mice had returned to resting state.

#### Gait Analysis

Gait analysis was tested using Catwalk™ (Noldus, The Netherlands), and carried out as previously described [7].

#### Food Pellet Taking

Skilled movements were assessed by testing food pellet handling. After food deprivation for 24 h, mice were videotaped to record food handling. IBB scores ranging from 0 to 9 were used to estimate forelimb usage, based on joint position, object support, digit movement, and grasping technique [14].

### Statistical Analysis

Results are presented as mean ± SEM, and the comparisons were done using unpaired Student’s *t*-test. *P* < 0.05 was noted as statistically significant.

## Results

### Knockout of *Celsr3* in the Brainstem Impairs Development of the RST

**Fig. 1:**
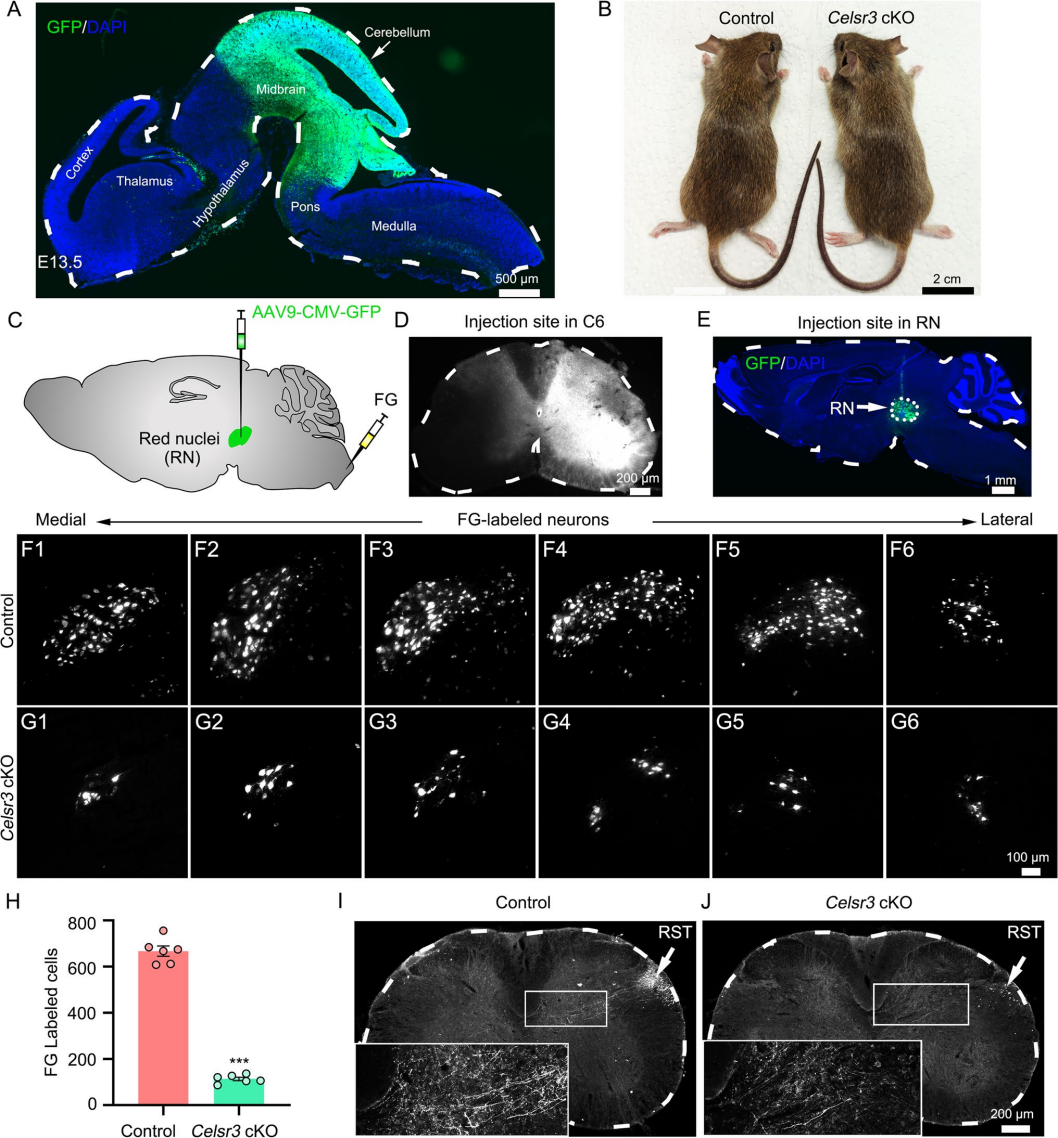
*Celsr3* cKO mice show impaired rubrospinal projections. (**A**) In E13.5 *En1-Cre;Rosa26*^*GFP*^ embryo, GFP positive signal is mainly distributed in midbrain and cerebellum. (**B**) Control and *Celsr3* cKO mice look similar. (**C–E**) Schema indicating AAV9-GFP (green) or FG (white) injection in red nuclei (RN) or C6 spinal segment, respectively (**C**). Injection sites are visualized in C6 spinal (**D**) and sagittal midbrain (**E**) sections. (**F–H**) FG-labeled RN neurons are visible in sagittal contralateral midbrain sections in the control (F1–F6) and the mutant (G1–G6). Quantification shows a significant decrease of FG-labeled neurons in the mutant compared to the control (**H**). ***P < 0.001; unpaired Student’s *t-*test; *n* = 6 in each group. (**I**, **J**) AAV9-GFP anterogradely labeled rubrospinal axons in sections of C5–C7 segments, in the control (**I**) and the mutant (**J**), showing many fibers in the control, but only scarce fibers in the mutant. RST, rubrospinal tract

Our previous studies validated Cre-loxP induced inactivation of “floxed” *Celsr3* gene [13, 15–18]. To indirectly monitor *Celsr3* inactivation upon En1-Cre expression, we generated *En1-Cre;Rosa26*^*GFP*^ mice. In sagittal sections of embryonic day (E) 13.5 brain, GFP signal was concentrated in the anlage of brainstem and cerebellum (Fig. 1A, *n* = 3). In the cerebellum, Celsr3 is expressed in Purkinje cells and *Celsr3* knockout affects motor learning but not walking ability in our recent report [19]. Here, we focused on inactivation of *Celsr3* in the brainstem. *En1-Cre;Celsr3*^*f/−*^ (*Celsr3* cKO) mice survived well and looked similar to littermate controls (Fig. 1B).

As En1-Cre is strongly expressed in the midbrain, we first studied RST projections in *Celsr3* cKO animals using anterograde and retrograde tracing. FG was injected into the right C6 spinal segment and retrogradely labeled neurons were counted in the left midbrain (Fig. 1C, D). AAV9-GFP was injected into left red nuclei and the anterogradely labeled fibers were observed in right halves of spinal cords (Fig. 1C, E). In serial parasagittal sections, FG-labeled red nuclei were readily identified in the midbrain in both control and mutant mice, but their number was dramatically reduced in the mutant (Fig. 1F1–F6, G1–G6). The total labeled red nuclei neurons were reduced by 83% in *Celsr3* cKO compared with the control (Fig. 1H; control and mutant: 667 ± 22 and 113 ± 7, *P* < 0.0001, Student’s *t*-test, *n* = 6 in each group).

Four weeks after AAV9-GFP injection into red nuclei (Fig. 1C, E), RST axons were visible in the superficial layer of dorsolateral white matter, and scattered in the gray matter in the control (Fig. 1I, *n* = 3), whereas rare GFP-labeled axons could be identified in mutant spinal segments (Fig. 1J, *n* = 3). In parasagittal sections of the brainstem, virus-transfected neurons were identified in red nuclei in two groups, but GFP-labeled neurons were reduced and less concentrated in *Celsr3* cKO mice (Supplementary Fig. 1). The impaired outgrowth of rubrospinal axons might result in neuronal death similar to the CST genetic absence [13], and affect the organization and/or location of red nuclei. In line with previous studies in other brain regions [13, 16], Celsr3 regulates the development of rubrospinal projections in a cell autonomous manner.

### Corticospinal, Vestibulospinal, and Propriospinal Projections Are Partially Reduced in *Celsr3* cKO Mice

**Fig. 2:**
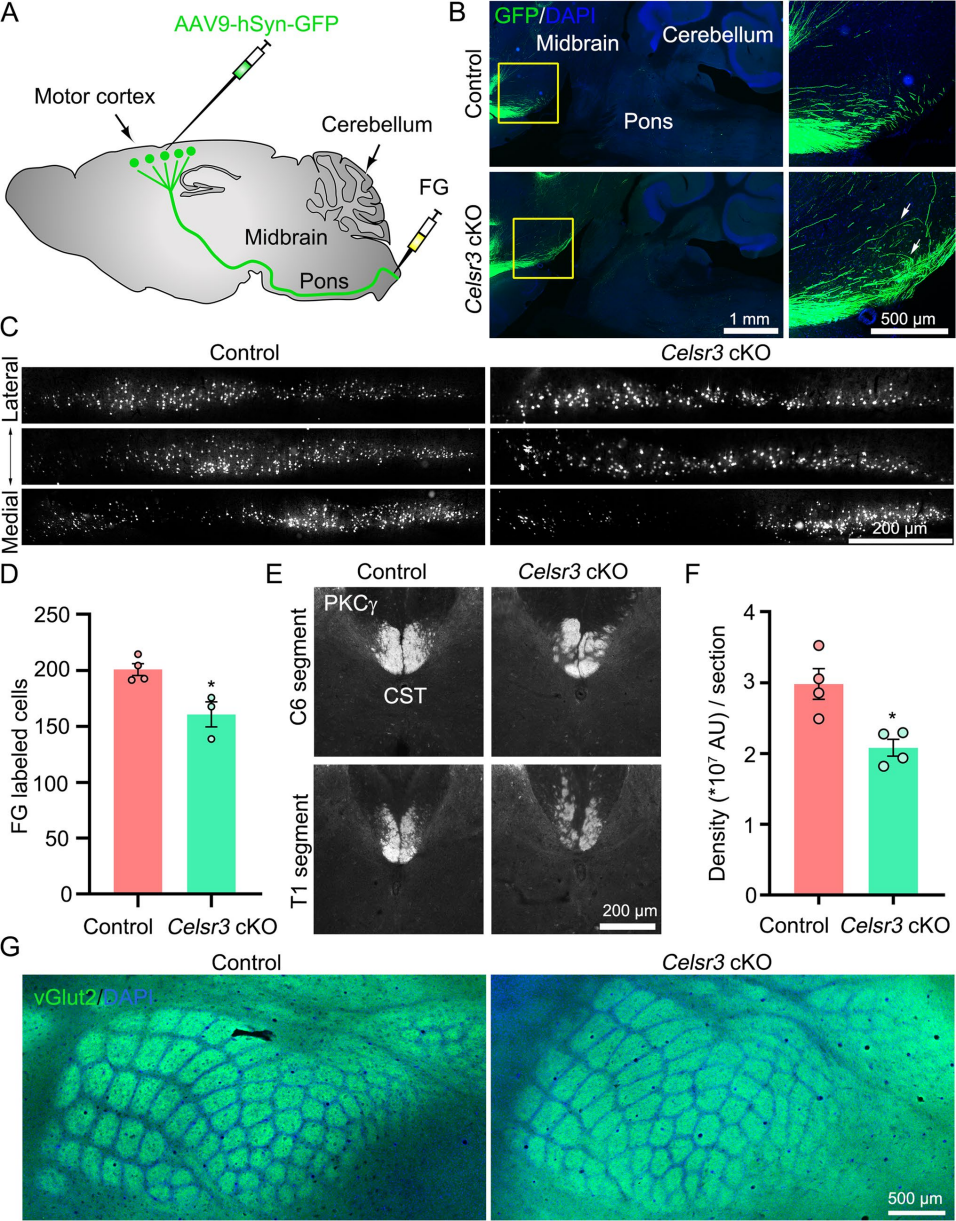
*Celsr3* cKO mice have defective corticospinal projections but normal organization of the barrel cortex. (**A**) Schematic drawing shows AAV9-hSyn-GFP virus (green) injection to retrogradely label CST axons and FG (white) injection to anterogradely label CST neurons. (**B**) In parasagittal sections, GFP-labeled CST are visible in the brainstem in both groups, and some axons are misrouted (arrows) in *Celsr3* cKO mice. Enlarged images in right panels are from selected areas in left panels respectively. (**C**,** D**) Injection of FG into C6 spinal segment retrogradely labeled corticospinal projecting neurons in sagittal sections of the cerebral cortex in the control and the mutant (**C**), with a decrease of neuron number in *Celsr3* cKO mice (**D**). *P < 0.05; unpaired Student’s *t*-test; *n* = 4 in the control and 3 in the mutant. (**E, F**) Anti-PKCγ immunostaining labels corticospinal fibers in the dorsal funiculus in C6 and T1 transverse spinal sections (**E**), with a reduction in *Celsr3* cKO mice (**F**). *P < 0.05; unpaired Student’s *t*-test, *n* = 4. (**G**) Anti-vGlut1 immunostaining discloses the organization of the barrel cortex without any difference between the control and the mutant

The brainstem is an important intermediate target for the pathfinding of corticospinal axons. To assess whether *Celsr3* inactivation impairs the development of the CST, we studied axon projections using anterograde tracing by injecting AAV9-hSyn-GFP virus into the motor cortex and retrograde tracing by injecting FG into C6 spinal segment (Fig. 2A). GFP-labeled CST axons were well visualized to travel through the brainstem in both groups, but some axons stopped and misrouted in the ventral region of the midbrain in *Celsr3* cKO mice (Fig. 2B). In FG tracing studies, retrogradely labeled neurons were found in layer V of contralateral parasagittal hemisphere sections, and displayed a laminar organization in the deep layer of the cortex (Fig. 2C), and their numbers were significantly reduced in the mutant compared to the control (Fig. 2D; control and mutant in cells/section: 201 ± 5 and 161 ± 11, *P* = 0.0168, Student’s *t*-test, *n* = 4 and 3 in the control and mutant respectively). In addition, we carried out anti-PKCγ immunostaining with C5–T1 spinal segments. In agreement with a previous report [20], PKCγ immunoreactivity disclosed CST descending axons in the dorsal funiculus in two groups, but axon bundles were disorganized in *Celsr3* cKO mice (Fig. 2E). The fiber density was decreased by 30% in the mutant compared to the control (Fig. 2F; control and mutant in 10^7^ pixels/section: 2.98 ± 0.22 and 2.08 ± 0.12, *P* = 0.0108, Student’s *t*-test, *n* = 4 in each group). These results show that Celsr3 in the brainstem regulates the development of the CST, in a non-cell autonomous manner.

Using FG retrograde tracing, we found a decrease of labeled neurons in the vestibular nuclei, but not in the pontine reticular nuclei in *Celsr3* cKO mice (Supplementary Fig. 2). Propriospinal projections were studied by CTB injection at C8–T1 segments, and retrogradely labeled neurons were counted in C3–C4 transverse spinal sections. The number was reduced on the ipsilateral side, but not on the contralateral side in *Celsr3* cKO mice (Supplementary Fig. 3).

The relay nuclei of trigeminal afferent pathways are located in the brainstem, and the pathway can be readily assessed by examining the organization of barrels in sensory cortex [21]. Adult hemispheres were prepared as flattened mounts and stained with anti-vGlut2. In primary somatosensory cortex, cortical barrels were identical in the mutant and the control (Fig. 2G), indicating that the development of the ascending sensory tract is not primarily dependent on Celsr3 expression in the brainstem.

### *Celsr3* cKO Mice Show Increased Branching of Dopaminergic Fibers in Spinal Segments

**Fig. 3:**
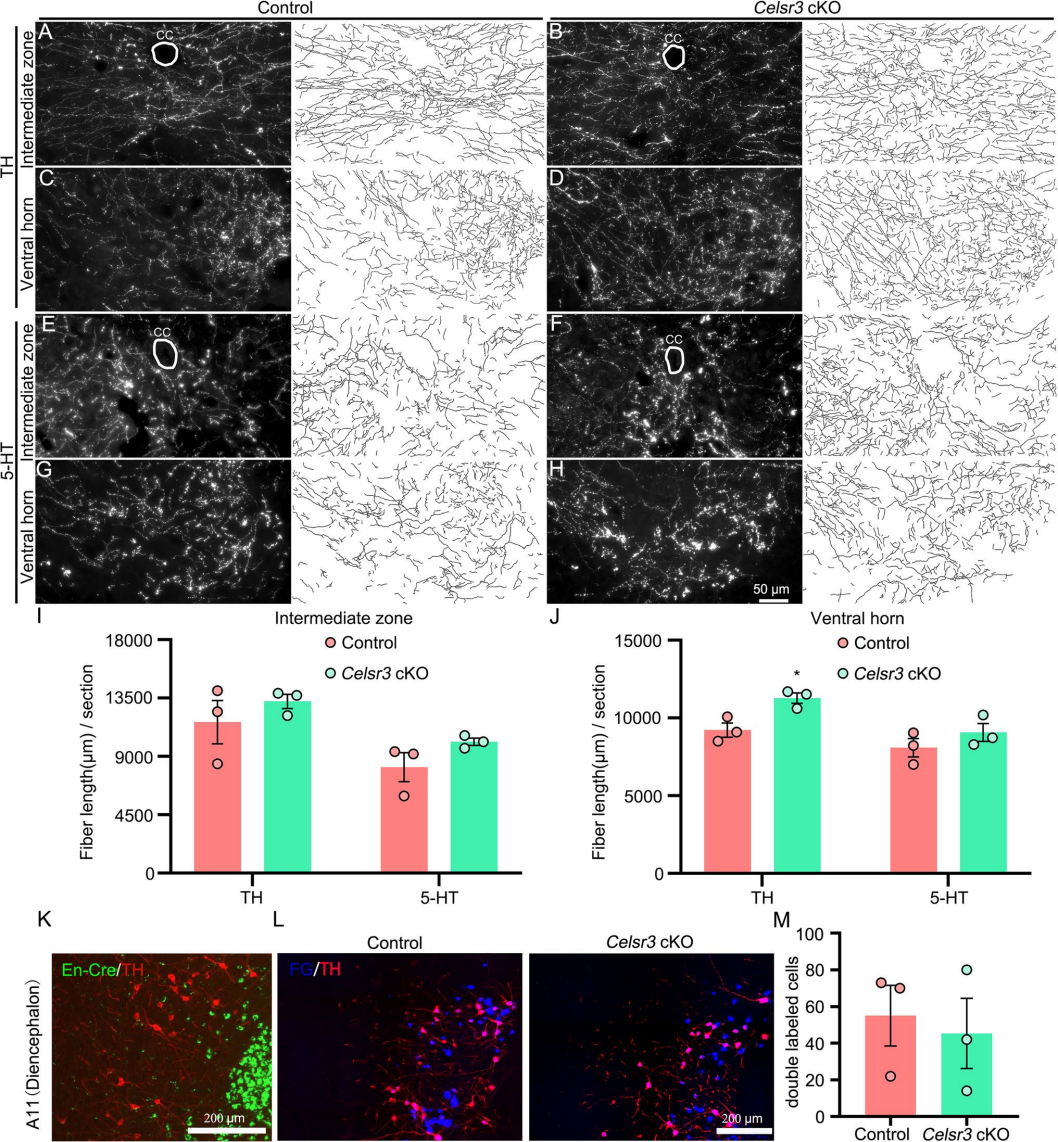
*Celsr3* cKO mice have increased branching of dopaminergic fibers in the spinal cord. (**A–D**) Anti-TH immunostaining of spinal sections (C8–T1 segments) indicates dopaminergic fibers in the intermediate zone and the ventral horn in the control (**A, C**) and the mutant (**B, D**). Reconstructed fibers are presented in the right panels beside each image. CC, central canal. (**E–H**) Serotoninergic fibers are visualized by anti-5HT immunostaining, with reconstructed images in the control (**E**, **G**) and the mutant (**F**, **H**). (**I**, **J**) *Celsr3* cKO mice have significantly increased the total length of dopaminergic fibers in the ventral horn, but not in intermediate zone. The length of serotoninergic fibers is similar in both groups. (**K**) Using *En1-Cre;Rosa26*^*GFP*^ mice, anti-TH immunostaining shows that dopaminergic neurons (red) do not co-express GFP in the A11 region of the diencephalon. (**L**, **M**) FG injection in spinal cords retrogradely labels projecting neurons (blue) in the A11 region, and dopaminergic neurons (red) are identified by anti-TH immunostaining (**L**). Statistic shows no differences of double-labeled neuron number in both groups (M). *P < 0.05; unpaired Student’s *t*-test, *n* = 3

Projections of dopaminergic axons from the diencephalon and serotonergic fibers from the raphe progress through the brainstem and innervate different spinal segments. Both are involved in motor control. We carried out anti-TH and -5-HT immunostaining of transverse spinal sections at C8–T1 segments and, performed tridimensional reconstruction of fibers (Fig. 3A–H). Intriguingly, in *Celsr3* cKO animals, the density of dopaminergic fibers was increased in the ventral horn (Fig. 3C, D), but not in the intermediate zone (Fig. 3A, B), compared to control animals (Fig. 3I; control and mutant in μm/section: 9222 ± 457 and 11,276 ± 336 in the ventral horn, 11,646 ± 1672 and 13,241 ± 549 in the intermediate zone, *P* = 0.0223 and 0.4161, respectively, Student’s *t*-test, *n* = 3 in each group). On the other hand, the density of serotoninergic fibers was comparable in both groups (Fig. 3E–H, J; control and mutant in μm/section: 8090 ± 591 and 9072 ± 574 in the ventral horn, 8169 ± 1116 and 10,123 ± 278 in the intermediate zone, *P* = 0.2991 and 0.644, respectively, Student’s *t*-test, *n* = 3 in each group).

Celsr3 is known to regulate dopaminergic axon pathfinding in a cell autonomous manner [22], and dopaminergic neurons projecting to spinal cord are mainly derived from diencephalic A11 region [23–25]. To try and understand the dopaminergic fiber phenotype in spinal segments of *Celsr3* cKO mutants, we studied Cre expression in dopaminergic neurons using *En1-Cre;Rosa26*^*GFP*^ mice combined with anti-TH immunostaining, and found no double-labeled neurons in the A11 region (Fig. 3K). This indicates that Celsr3 expression is preserved in spinal-projecting dopaminergic neurons in *Celsr3* cKO mice. Using FG retrograde labeling combined with TH immunostaining of sagittal brain sections, FG-labeled neurons in A11 were positive for TH and present in comparable numbers in mutant and control samples (Fig. 3L, M; control and mutant in cells/section: 55 ± 17 and 45 ± 19, *P* = 0.7216, Student’s *t*-test, *n* = 3 in each group). These results suggest that increased dopaminergic fiber density in *Celsr3* cKO animals is due to compensatory axonal branching when the CST and RST are defective.

### Maturation and Output of Spinal Motoneurons Are Impaired in *Celsr3* cKO Mice

**Fig. 4:**
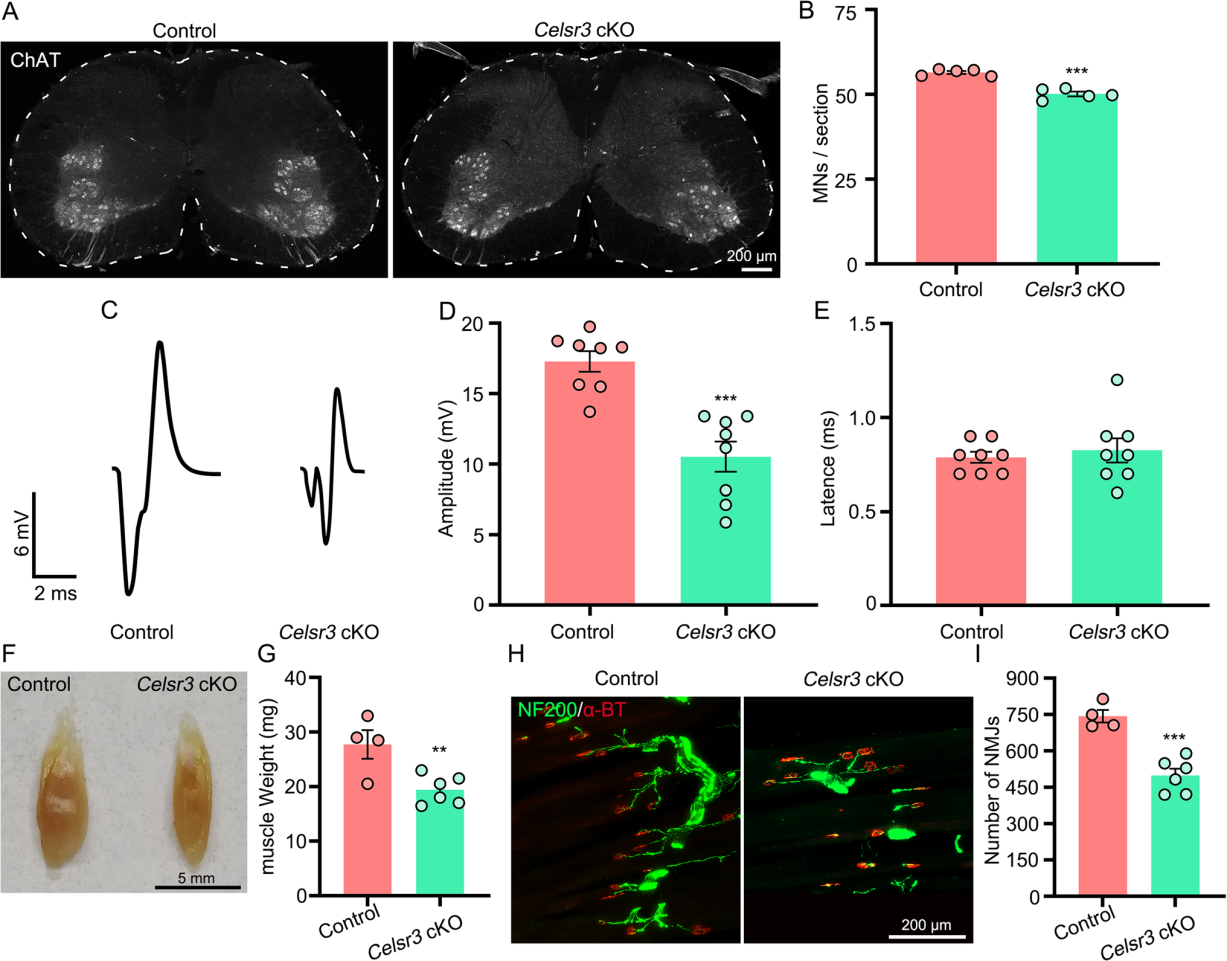
*Celsr3* cKO mouse shows defective motor maturation. (**A**, **B**) In C5–C8 spinal segments, ChAT immunostaining (**A**) shows a significant decrease of spinal motor neurons in *Celsr3* cKO mice (**B**). ***P < 0.001; unpaired Student’s *t*-test; *n* = 5. (**C–E**) EMG recording of biceps (**C**) shows an increase of peak-to-peak amplitude (**D**), but a comparable latency (**E**) in *Celsr3* cKO mice compared with control mice. ***P < 0.001; unpaired Student’s *t*-test; *n* = 8. (**F**, **G**) In *Celsr3* cKO mice, biceps are hypotrophic (**F**) with a significant decrease of wet weight compared with control mice (**G**). **P < 0.01; unpaired Student’s *t*-test; *n* = 4 in the control and 6 in the mutant. (**H**, **I**) *Celsr3* cKO mice show decreased NMJs in the biceps, visualized by anti-NF200 and α-BT staining, compared to control mice. ***P < 0.001; unpaired Student’s *t*-test, *n* = 4 in control, and 6 in mutant

The activity of spinal motoneurons is driven by corticospinal, rubrospinal, and propriospinal inputs, mainly through indirect connections in adult rodents [26]. To test whether defects of these descending tracts impact motoneuron maturation and function in the mutant, transverse spinal sections at C5–T1 were prepared for anti-ChAT immunostaining (Fig. 4A). The number of spinal motoneurons was subtly decreased in the mutant compared to the control (Fig. 4B; control and mutant in cells/section: 56.5 ± 0.4 and 50.1 ± 0.7, *P* < 0.0001, Student’s *t*-test, *n* = 5 in each group). Upon stimulation of musculocutaneous nerves, the EMG recording of biceps showed a significant reduction in amplitude, but not of latency, in *Celsr3* cKO mice compared to control mice (Fig. 4C–E; control and mutant: 17.3 ± 0.7 and 10.5 ± 1.1 mV in amplitude, 0.79 ± 0.03 and 0.83 ± 0.06 ms in latency; *P* = 0.0001 and 0.6066 respectively, Student’s *t*-test, *n* = 8 in each group). The mutant biceps were hypotrophic, with a significant reduction of wet weight compared to controls (Fig. 4F, G; control and mutant in mg: 27.8 ± 2.6 and 19.4 ± 1.1, *P* = 0.0097, Student’s *t*-test, *n* = 4 and 6 in the control and the mutant). We studied NMJs by staining for NF200 (fiber terminus) and α-BT (acetylcholine receptors) in biceps, and found a 33% of reduction in the mutant compared to the control (Fig. 4H, I; control and mutant in NMJs/muscle: 743 ± 26 and 499 ± 28, *P* = 0.0003, Student’s *t*-test, *n* = 4 and 6 in the control and the mutant).

### *Celsr3* cKO Mice Have Defective Motor Coordination but Normal Walking Ability and Fine-Movement Control

**Fig. 5:**
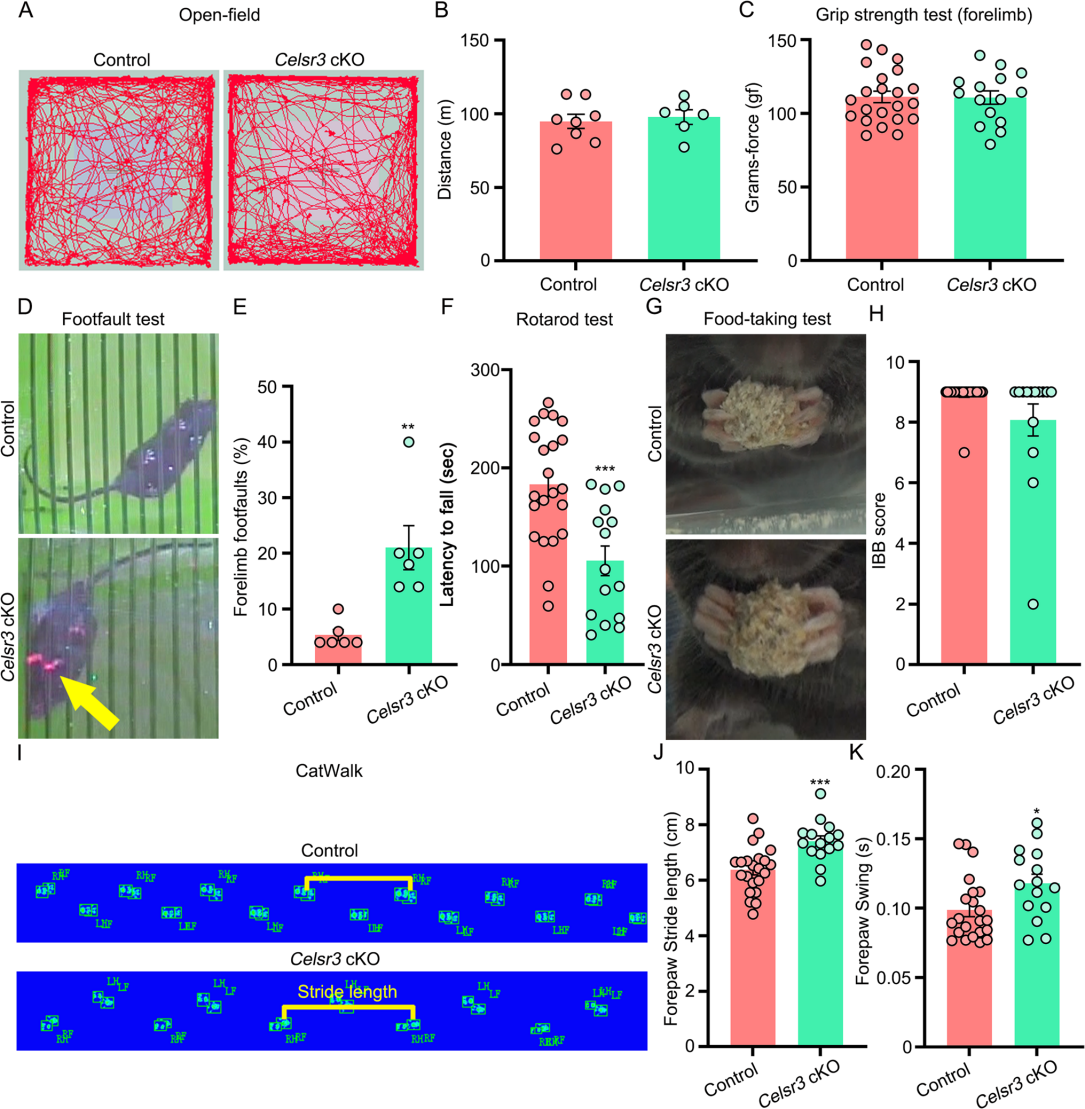
*Celsr3* cKO mice have deficits of motor coordination. (**A, B**) Open-field tests show no significant differences of movement trajectory (**A**) and 15-min moving distance (**B**) between both groups. *P* > 0.05; unpaired Student’s *t*-test; *n* = 8 in the control and 6 in the mutant. (**C**) Grip strength of forelimbs is comparable in the two groups. *P* > 0.05; unpaired Student’s *t*-test; *n* = 22 in the control and 15 in the mutant. (**D**,** E**) *Celsr3* cKO mice show an increase of footfaults compared to control mice. **P < 0.01; unpaired Student’s *t*-test; *n* = 6. (**F**) The latency of falling during the accelerating Rotarod is significantly decreased in *Celsr3* cKO mice. ***P < 0.001; unpaired Student’s *t*-test; *n* = 22 in the control and 15 in the mutant. (**G**, **H**) In the food pellet taking test (**G**), the IBB scores are comparable in both groups (**H**). *P* > 0.05; unpaired Student’s *t*-test; *n* = 23 and 14 in the control and the mutant. (**I–K**) Catwalk tests show a significant decrease of forepaw stride length (**J**) and swing in the *Celsr3* cKO. *P < 0.05; ***P < 0.001; unpaired Student’s *t*-test; *n* = 22 in the control and 15 in the mutant

As described above, various descending axonal tracts were modified in *Celsr3* cKO mice. To assess the consequences in terms of motor behavior, we studied locomotion and fine movement control. In open-field tests, there were no significant differences of movement trajectory and total distance between control and mutant mice (Fig. 5A, B; control and mutant in m: 94.8 ± 4.8 and 97.8 ± 5.0, *P* = 0.6802, Student’s *t*-test, *n* = 8 and 6 in the control and the mutant). The grip strength of forelimbs was similar in both groups (Fig. 5C; control and mutant in gf: 111.2 ± 3.9 and 110.8 ± 4.5, *P* = 0.9376, Student’s *t*-test, *n* = 8 and 6 in the control and the mutant). In contrast, the percentage of footslips in grid tests was significantly increased in *Celsr3* cKO compared to controls (Fig. 5D, E; control and mutant: 5.33 ± 0.99% and 21.00 ± 3.96%, *P* = 0.0033, Student’s *t*-test, *n* = 6 in each group). In the Rotarod test, *Celsr3* cKO mice had a shorter falling latency than control mice (Fig. 5F; control and mutant in sec: 183.3 ± 12.5 and 105.5 ± 14.9, *P* = 0.0003, Student’s *t*-test, *n* = 22 and 15 in the control and the mutant). Unexpectedly, the IBB scores in food pellet taking were comparable in both groups (Fig. 5G, H; control and mutant: 8.91 ± 0.09 and 8.07 ± 0.53, *P* = 0.055, Student’s *t*-test, *n* = 23 and 14 in the control and the mutant), indicating that skilled movement is not affected in the mutant. The voluntary gait, assessed using the Catwalk (Fig. 5I), showed a longer forepaw stride (Fig. 5J; control and mutant in cm: 6.37 ± 0.18 and 7.41 ± 0.19, *P* = 0.0004, Student’s *t*-test, *n* = 22 and 15 in the control and the mutant), and a significant increase of forepaw swing in *Celsr3* cKO compared to control mice (Fig. 5K; control and mutant in s: 0.099 ± 0.005 and 0.118 ± 0.007, *P* = 0.0215, Student’s *t*-test, *n* = 23 and 14 in the control and the mutant).

### Response to Strong Mechanical Stimulation Is Impaired in *Celsr3* cKO Mice

**Fig. 6:**
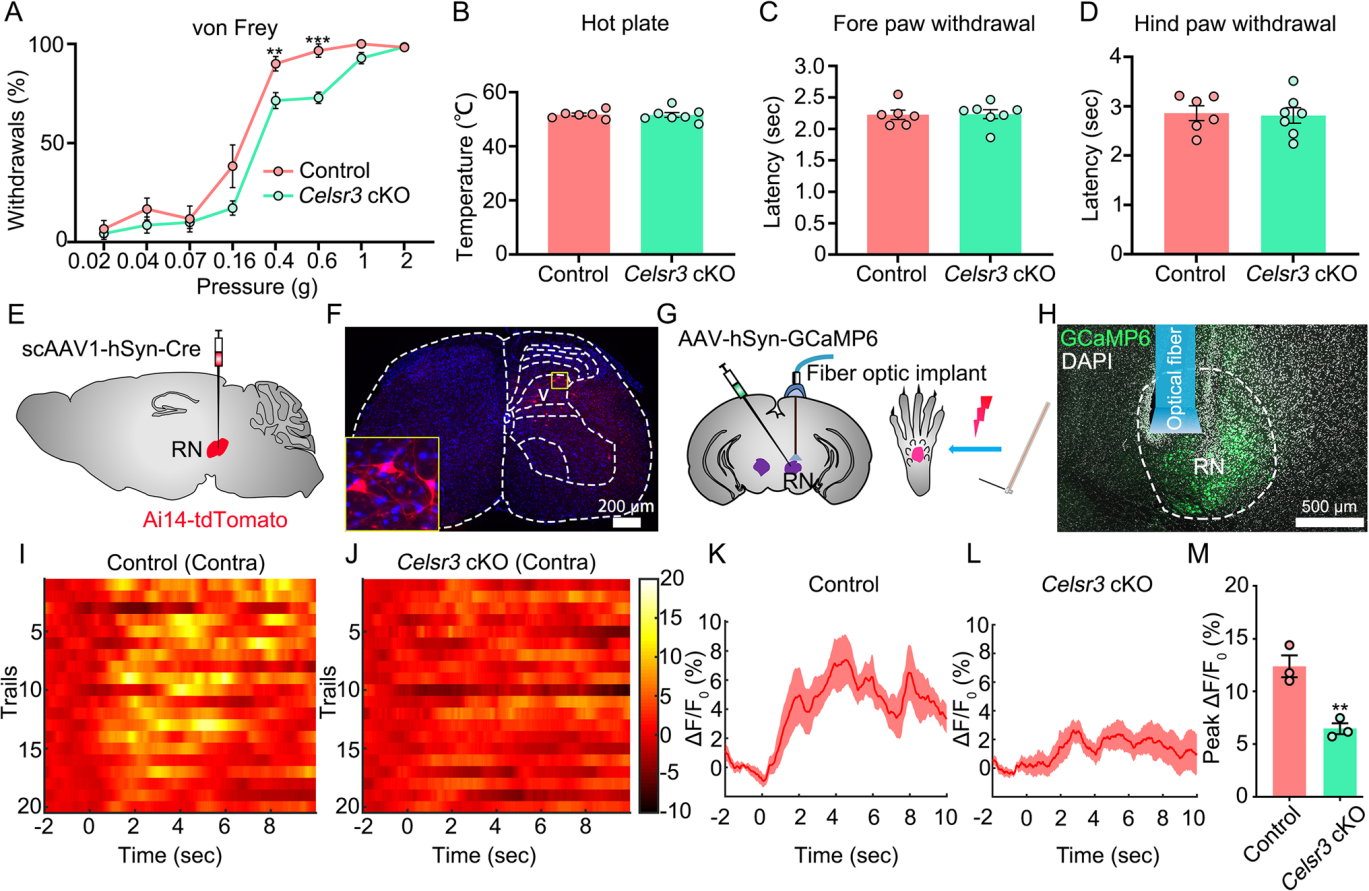
*Celsr3* cKO mice show abnormal response to heavy mechanical stimulation. (**A**) In the von Frey test, *Celsr3* cKO mice are less sensitive to the stimulation of fiber stiffness at 0.4 g and 0.6 g compared to control mice. **P < 0.01; ***P < 0.001; unpaired Student’s *t*-test; *n* = 6 in the control and 7 in the mutant. (**B**–**D**) Hot plate tests show the similar temperature threshold (**B**), and no differences of latencies of forepaw (**C**) or hindpaw withdrawal in the two groups. *P* > 0.05; unpaired Student’s *t*-test; *n* = 6 in the control and 7 in the mutant. (**E**, **F**) In Ai14-tdTomato mice, injection of scAAV1-hSyn-Cre into the red nuclei (RN, **E**) labels transsynaptically spinal neurons (red) in the dorsal horn (**F**). (**G**–**L**) Schematic drawing illustrating injection of AAV-hSyn-GCaMP6 and implantation of an optic fiber in the RN, and stimulation of mouse hindpaw (**G**). Upon pinprick stimulation, the calcium signal of contralateral (contra) RN neurons is recorded (**H**). The heatmaps show the stronger response in the control (**I**) than that in the mutant (**J**), as indicated in the histogram of time-dependent averaged calcium signal respectively (**K, L**). Quantitative analysis shows a significant increase of the calcium signal peak in the mutant compared to that in the control (**M**). **P < 0.01; unpaired Student’s *t*-test; 20 trials from 3 mice in each group

Recent studies showed that descending projections from the cortex modulate sensory processing in spinal cord [8]. We wondered whether any sensory abnormalities are present in *Celsr3* cKO mutants, and carried out pain, thermal, and mechanical sensation tests in adult animals. Interestingly, in von Frey tests, the percentage of hindpaw withdrawal was significantly decreased with stimuli at 0.4 g and 0.6 g stiffness in *Celsr3* cKO mice compared to control mice (Fig. 6A; control and mutant: 90 ± 4% and 71 ± 4% at 0.4 g, 97 ± 3 and 73 ± 3 at 0.6 g, *P* = 0.0064 and 0.0002, Student’s *t*-test, *n* = 6 and 7 in the control and the mutant), indicating an impairment of mechanical sensation in the mutant. Upon laser stimulation of paws, the withdrawal latencies of forepaws and hindpaws showed no differences between both groups (Fig. 6B–D; control and mutant: 51.7 ± 0.6 and 51.6 ± 0.9 °C for hot plate, 2.24 ± 0.07 and 2.23 ± 0.07 s for forepaws, 2.86 ± 0.15 s and 2.81 ± 0.16 s for hindpaws, *P* = 0.9124, 0.9242 and 0.8392, Student’s *t*-test, *n* = 6 and 7 in the control and the mutant).

In order to know whether the RST is involved in the modulation of nociceptive mechanical perception, we studied the synaptic connections of rubrospinal axons with spinal neurons by injecting transsynaptic scAAV1-hSyn-Cre virus into the red nuclei of Ai14-tdTomato mice (Fig. 6E), and found that Tomato-positive spinal neurons were scattered in layers V–VII of the contralateral gray matter (Fig. 6F), suggesting that rubrospinal axons directly synapse on spinal neurons in the deep layer of the dorsal horn. Upon mechanical stimuli on hindpaws, calcium activities of contralateral red nuclei were recorded (Fig. 6G, H). The calcium signal was strong in controls (Fig. 6I, K), but less prominent in *Celsr3* cKO mutants (Fig. 6J, L). Quantitative analysis showed a significant decrease of calcium signal peak in the mutant compared to the control (Fig. 6M; control and mutant in ΔF/F: 12.39 ± 1.03% and 6.47 ± 0.53%; *P* = 0.007, Student’s *t*-test, *n* = 3). This suggests that the RST is required for the response to mechanical stimulation.

## Discussion

The brainstem is traversed by longitudinal descending and ascending axonal tracts that connect the telencephalon and the spinal cord. In addition, red nuclei, vestibular nuclei, and reticular nuclei located in the brainstem send projections to different spinal segments involved in motor control and possibly in sensory modulation. Conditional inactivation of *Celsr3* has been widely used to study axonal projections in forebrain and hippocampus [13, 15, 16]. Here, we inactivated *Celsr3* in the brainstem using En1-Cre, and found that this resulted in a drastic reduction of the RST. We observed abnormalities of motor coordination and of response to heavy mechanical stimulation. Mutant animals also showed a slight reduction of corticospinal, vestibulospinal, and propriospinal projections, but an increase of dopaminergic axon branching in spinal segments.

*Celsr3* is a mammalian orthologue of *Drosophila flamingo*. Its mRNA is highly expressed in the central nervous system during embryonic development and downregulated after birth [27]. Upon Cre recombination driven by En1, which is expressed in the midbrain at early developmental stages [28], *Celsr3* is inactivated prior to axonal extension. In *Celsr3* cKO mutants, most rubrospinal axons (more than 80%) fail to reach spinal segments, indicating that Celsr3 expression in rubrospinal neurons is indispensable for RST formation, in a cell autonomous manner. Intriguingly, the CST is also partially reduced (about 30%) in spinal cord, even though Celsr3 expression in cortical neurons is not affected in *Celsr3* cKO mice. At least two possibilities may explain this non-cell autonomous CST phenotype: (i) Celsr3-expressing cells in the brainstem could similarly function as guidepost cells to steer corticospinal axons as reported in the forebrain [13, 15], which is supported by the finding that some CST axons are misrouted at the midbrain in the mutant; (ii) the rubrospinal and corticospinal axons develop in concert, and the reduced corticospinal axons in the spinal cord are a consequence of the impaired rubrospinal projections. These results indicate that Celsr3 steers axon wiring in both cell and non-cell autonomous manners. The mechanism may also explain the phenotype of vestibulospinal and propriospinal projections in *Celsr3* cKO mice.

In *Celsr3* cKO mutants, the expression of Celsr3 may be affected in dopaminergic neurons in the midbrain [29], but not in the A11 region, which is the origin of spinal-projecting dopaminergic neurons, confirmed by lineage tracing and TH immunostaining. Dopaminergic axons project normally to spinal segments, and axon branching shows compensatory increase particularly in the ventral horn in *Celsr3* cKO animals. These findings further indicate that different descending inputs to spinal motor neurons interact during neural circuit development and maturation, and that those interactions remain largely unknown.

Celsr3 regulates growth of spinal motor neuron axons in a cell autonomous manner [30]. Our observations of alterations in spinal motor neurons, NMJs, and EMG amplitude in biceps show that Celsr3 also impacts motor neuron indirectly. These results suggest that the maturation of spinal motor network is highly activity dependent, in agreement with the previous reports [7, 31]. However, in adult rats, the unilateral rubrospinal tract transection did not affect the survival of spinal motor neurons [32]. Therefore, descending inputs are critical for maintaining the appropriate number of spinal motor neurons and their muscle targeting during development, and the interruption of descending tracts has less impact on spinal motor neuron survival, once the motor network is established.

Although *Celsr3* cKO mice have a dramatic reduction of rubrospinal projections (more than 80%), a partial reduction in corticospinal axons (about 30% decrease), and an impairment of NMJs, their only motor deficit is in poor coordination (Catwalk and Rotarod tests). Mutant animals have normal walking in the open-field test and unexpectedly preservation of skilled, fine motor control during food pellet handling. The phenotype is strikingly different from that in the mice with a complete genetic absence of the CST [7], suggesting that, although the CST and RST work in concert at least in mice [33], fine forelimb and digit control is highly dependent on commands from the motor cortex, but not the red nuclei. Alternatively, the fine motor control might be compensated during development in *Celsr3* cKO mutants, and our finding is different from previous reports: the RST or red nuclei lesion in adulthood impairs arpeggio movements or skilled distal forelimb function in rats [34, 35].

Lastly, although the main function of the RST is in maintenance of body posture and movement coordination [36], we found that *Celsr3* cKO mice had impaired response to heavy mechanical stimulation (0.4 and 0.6 g). In contrast, defects of the CST mainly result in an abnormal response to light mechanical stimulation (0.16 g or brush stimulation) [8]. Our findings indicate that the RST is involved in the mechanical sensory process. Using calcium neuronal activity recording, we show that strong mechanical stimulation of hindpaws activates red nuclei, and that this is defective in *Celsr3* cKO mice. Our results are also supported by previous reports. In cats, a direct ascending spino-rubral projection conveys somatosensory information to the red nuclei [37, 38], and stimulation of red nuclei can inhibit nociceptive neurons in the spinal dorsal horn [39, 40]. Using task-related functional imaging in humans, passive tactile stimulation of the finger pads provokes red nucleus activation [10]. Altogether, the rubrospinal system is an essential component of sensory and motor feedback loops in different species, and this is critical for sensorimotor integration.

## Supplementary Information

Below is the link to the electronic supplementary material.

Supplementary file1 (DOCX 3681 KB)

## Data Availability

The data that support the findings of this study are available from the corresponding author upon reasonable request.
