# Supplementary material for: Celsr3 Inactivation in the Brainstem Impairs Rubrospinal Tract Development and Mouse Behaviors in Motor Coordination and Mechanic-Induced Response
Source: Mol Neurobiol. 2022 Jun 9;59(8):5179–92. doi: 10.1007/s12035-022-02910-7 (PMC9363480; doi:10.1007/s12035-022-02910-7)
Supplement: Supplementary file 1 — Supplementary file1 (DOCX 3681 KB) [file 12035_2022_2910_MOESM1_ESM.docx]

Supplementary Materials

*Celsr3* inactivation in the brainstem impairs rubrospinal tract development and mouse behaviors in motor coordination and mechanic-induced response

Boli Chen^1^, Fuxiang Li^1^, Bin Jia^1^, Kwok-Fai So^1, 2, 3, 4, 5, 6^, Ji-An Wei^7^, Yuchu Liu^7^, Yibo Qu^1^, Libing Zhou^1, 2, 3, 4, 5, 6*^


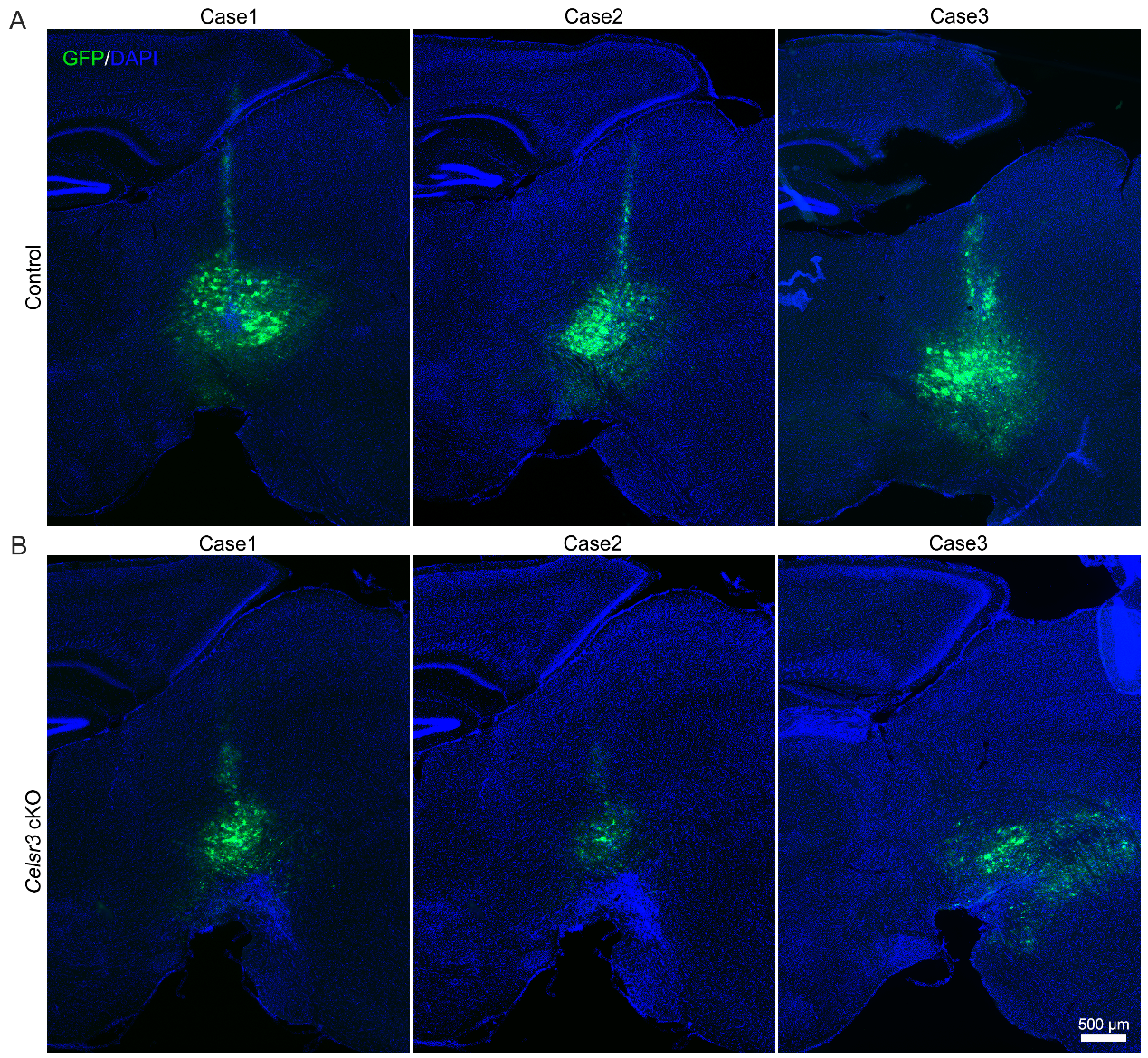


Supplementary Figure 1. AAV9-GFP transfected neurons are visualized in the midbrain.

AAV9-GFP viruses are injected into red nuclei in 3 control (A) and 3 *Celsr3* cKO mice (B). In parasagittal sections of the brainstem, GFP-labeled red nuclei were reduced and less concentrated in *Celsr3* cKO mice.


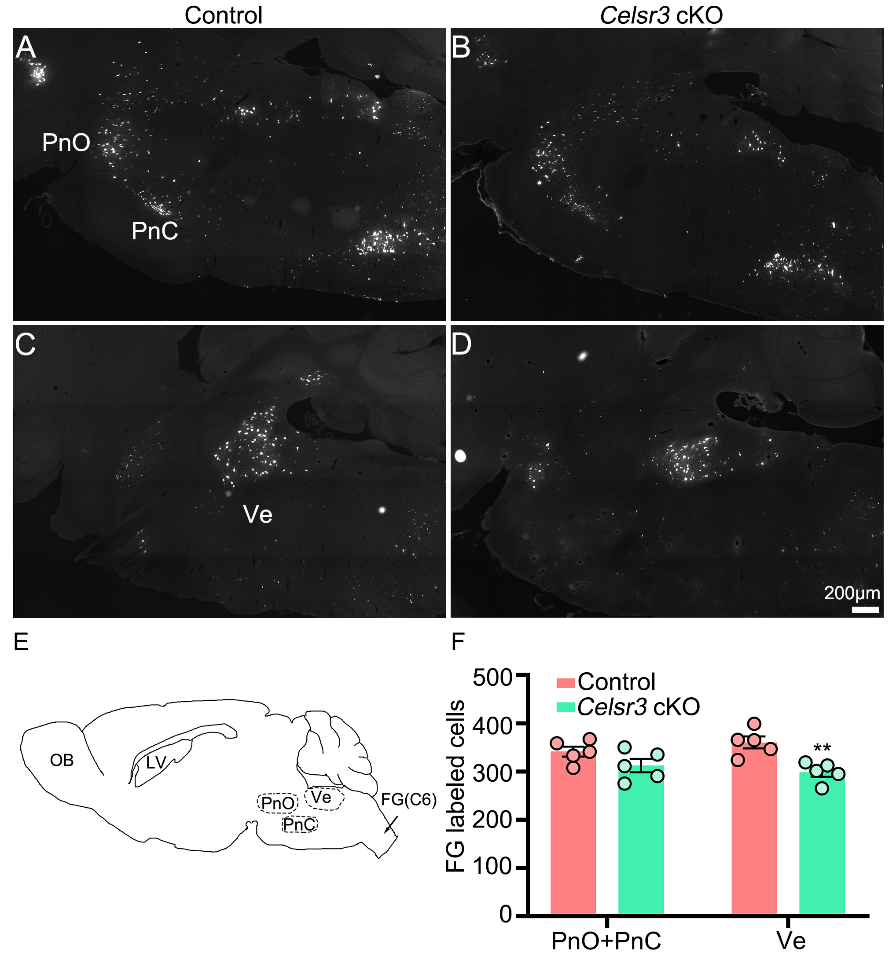


Supplementary Figure 2. Vestibulospinal projections are reduced in *Celsr3* cKO mice.

A-D: FG retrogradely labels caudal pontine reticular nuclei (PnC), oral pontine reticular nuclei (PnO) and vestibular nuclei (Ve) in control and *Celsr3* cKO mice.

E: Schema indicating FG injection at C6 segment.

F: Statistics show a decrease of FG-labeled neurons in the vestibular nuclei in *Celsr3* cKO mice, but no differences in the PnC and PnO between two groups. **, *P<*0.01; unpaired Student’s *t*-test, n=5.


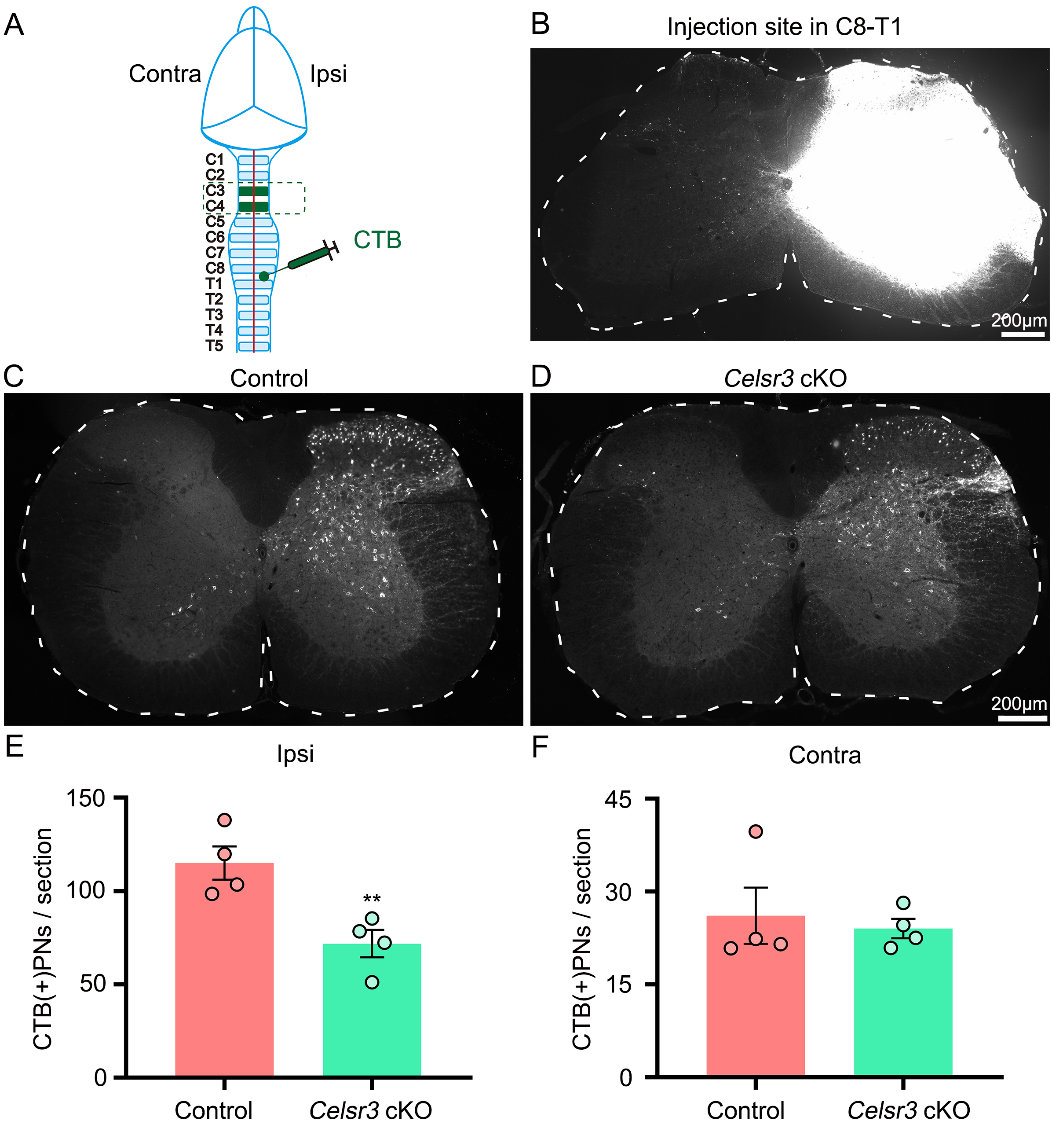


Supplementary Figure 3. Ipsilateral propriospinal projections are decreased in *Celsr3* cKO mice.

A: Schema shows CTB injection in right C8–T1 segments and labeled neurons at C3-C4 segments for analysis. Contra, contralateral side; Ipsi, ipsilateral side.

B: Transverse spinal section indicates the injection site at C8–T1 segments.

C-D: CTB-labeled propriospinal neurons (PNs) are visible at C3-C4 segments, more on the ipsilateral side in both groups.

E-F: Statistics show a decrease of CTB-labeled PNs in *Celsr3* cKO mice on the ipsilateral side, but no differences on the contralateral side. **, *P<*0.01; unpaired Student’s *t*-test, n=4.
